# Self-Rectifying Resistive Switching and Short-Term Memory Characteristics in Pt/HfO_2_/TaO*_x_*/TiN Artificial Synaptic Device

**DOI:** 10.3390/nano10112159

**Published:** 2020-10-29

**Authors:** Hojeong Ryu, Sungjun Kim

**Affiliations:** Division of Electronics and Electrical Engineering, Dongguk University, Seoul 04620, Korea; hojeong.ryu95@gmail.com

**Keywords:** resistive switching, memristor, self-rectifying, synaptic device, short-term memory, neuromorphic system

## Abstract

Here, we propose a Pt/HfO_2_/TaO*_x_*/TiN artificial synaptic device that is an excellent candidate for artificial synapses. First, XPS analysis is conducted to provide the dielectric (HfO_2_/TaO*_x_*/TiN) information deposited by DC sputtering and atomic layer deposition (ALD). The self-rectifying resistive switching characteristics are achieved by the asymmetric device stack, which is an advantage of the current suppression in the crossbar array structure. The results show that the programmed data are lost over time and that the decay rate, which is verified from the retention test, can be adjusted by controlling the compliance current (CC). Based on these properties, we emulate bio-synaptic characteristics, such as short-term plasticity (STP), long-term plasticity (LTP), and paired-pulse facilitation (PPF), in the self-rectifying I–V characteristics of the Pt/HfO_2_/TaO*_x_*/TiN bilayer memristor device. The PPF characteristics are mimicked by replacing the bio-stimulation with the interval time of paired pulse inputs. The typical potentiation and depression are also implemented by optimizing the set and reset pulse. Finally, we demonstrate the natural depression by varying the interval time between pulse inputs.

## 1. Introduction

Resistive random-access memory (RRAM) based on various metal oxides has been extensively studied due to its superior non-volatile memory performance, specifically in terms of its high endurance, low-power operation, high speed switching, and good complementary metal-oxide-semiconductor (CMOS) compatibility [1,2,3,4,5,6,7,8,9,10]. Metal oxides, such as TaO*_x_* and HfO*_x_*, have better reproducibility and variability than other material systems like organic [11] and 2D materials [12]. The biggest advantage of RRAM for high-density memory with 4F^2^ (F is feature size) is that only two metal lines (two-terminal) are needed to access the memory cells. The cross-point arrangement of memory cells allows for RRAM and phase change random-access memory (PRAM) [13] to be more highly integrated than other emerging memory devices such as magnetic random-access memory (MRAM) [14] and ferroelectric random-access memory (FRAM) [15]. Further, the crossbar array structure has the highest memory density when a 3D vertical stack is used [16]. The half-bias scheme is commonly used for reading and writing operations on the word line and the bit line. However, the read error can occur when only resistors with linear I–V characteristics are located at the intersection of the word line and the bit line [17]. An unintentional leakage current is generated from the neighboring cells since multiple cells are connected through the bit line and the word line. In addition, leakage current flows through the unselected cells in a low resistance state (LRS) when accessing the data on the target memory cell. Therefore, there is an urgent need for a solution which prevents the sneak current problem in the crossbar array. To overcome the sneak current limitation in the 1R (resistor) structure, a 1S1R (1 selector + 1 resistor) structure has been presented. However, it is not suitable for a bipolar type resistor when a diode that only provides the unidirectional selection function is connected to each resistor. It is also difficult to implement a multi-layered 3D structure due to difficulties in the fabrication process. When merging the resistor with a metal-insulator-metal (MIM) type selector, the integration processes and the fitting of the operation voltage are not easy [18]. In an attempt to eliminate the sneak current while using an easy fabrication process, the self-rectifying RRAM has been presented. It has an inherent rectification function, so its device stack is simple. This means that there is no need for an additional selector for a resistor in the crossbar array. As a result, it can be useful in a high-density crossbar array structure. Further, a silicon substrate with moderate impurity can provide the self-rectifying feature, but line resistance could be a serious drawback [19,20,21,22]. 

The typical structure of a self-rectifying RRAM is metal-insulator-insulator-metal (MIIM). The large work function difference between the top and bottom electrodes is essential for the asymmetric effective barrier seen in the top and bottom electrodes. In addition, one in two insulator layers is maintained for the insulating property in the LRS. To date, several self-rectifying RRAM structures have been proposed, such as Pt/Ta_2_O_5_/HfO_2−*x*_/TiN [23], Ni/SiN/HfO_2_/Si [24], and Pd/HfO_2_/TaO*_x_*/Ta [25]. However, there have been few papers that have thoroughly verified whether a device has the volatile or non-volatile property. Recently, many researchers have investigated using RRAM as a synaptic device for a neuromorphic system [26,27,28]. The von Neumann architecture struggles with data processing in the current big data era due to the bottleneck that arises between memory and computing, so new computing systems such as the neuromorphic system have been introduced to save computing energy and to cope with difficult tasks such as pattern recognition [29]. The first step is to try to understand and emulate biological synapses for implementation in neuromorphic systems. The human brain maintains memory through two types of synaptic plasticity: short-term plasticity (STP) and long-term plasticity (LTP). For on-chip learning in a neuromorphic system, the higher the number of conductance, the better, and the update of conductance is preferably linear and symmetric [30].

Here, we mimic the synaptic functions of STP and LTP in Pt/HfO_2_/TaO*_x_*/TiN memristor devices. Bio-synaptic simulation is emulated in RRAM devices by controlling the input pulse repetition and the interval time. The frequency of the pulse can alter the strength of the filament in the insulator of an RRAM device. This is likened to the process through which the human brain remembers and forgets information. The input of intermittent pulses forms a thin filament. After the stimulus is removed, it causes a spontaneous collapse, called STP. On the other hand, in high frequency stimulus, the filament does not collapse easily even if the stimulus is removed, which is called LTP. Such devices also use synaptic plasticity features that mimic human memory characteristics.

In this paper, we study the self-rectifying and multi-level conductance characteristics of an HfO_2_/TaO*_x_* bilayer RRAM device. The HfO_2_/TaO*_x_* bilayer stack has already been reported for non-volatile memory applications. However, there have been few reports on the simultaneously intrinsic self-rectifying and volatile characteristics with artificial synaptic properties for neuromorphic computers. This paper mainly demonstrates long-term plasticity and short-term plasticity in the inherent self-rectifying I–V behaviors of the Pt/HfO_2_/TaO*_x_*/TiN device. The retention properties are investigated at different current levels for STM. The gradual conductance modulations are implemented using two methods (polarity and pulse frequency control). Moreover, the PPF characteristics are conducted to mimic the neurodynamic properties of the human brain, such as STP.

## 2. Materials and Methods

The Pt/HfO_2_/TaO*_x_*/TiN memristor device was fabricated on a square SiO_2_/Si wafer substrate. First, a 100 nm thick TiN was deposited as the bottom electrode by DC sputtering on SiO_2_/Si substrate. For the HfO_2_/TaO*_x_* bilayers, a 20 nm thick TaO*_x_* thin film was deposited by pulsed DC reactive sputtering. The substrate temperature was room temperature, the base pressure was 1.6 × 10^−6^ Torr, and the gas flow rates of Ar and O_2_ were 8 sccm and 12 sccm, respectively. The deposition pressure was 1 mTorr, and the DC power (pulsed DC, 50 kHz) was 500 W. Then, HfO_2_ was deposited as the second insulator by atomic layer deposition (ALD). The internal temperature was 280 °C and the source temperature was 90 °C. The composition of one cycle of HfO_2_ is as follows: TEMAHf 0.5 s/purge 35 s/H_2_O 0.3 s/purge 35 s; 105 cycles were performed for deposition of the target thickness of 7 nm. Finally, a shadow mask with a diameter of 100 µm was covered, and 100 nm of Pt was deposited as the top electrode on HfO_2_ using e-beam evaporation. All electrical characteristics, such as DC sweep mode and pulse mode, were investigated with a semiconductor parameter analyzer (Keithley 4200-SCS and 4225-PMU ultrafast module). During all the measurements, the bias and pulse voltages were applied to the top electrode, Pt, while the bottom electrode, TiN, was grounded. X-ray photoelectron spectroscopy (XPS) depth analysis was conducted, using a Nexsa (ThermoFisher Scientific, Waltham, MA, USA) with a Microfocus monochromatic X-ray source (Al-Kα (1486.6 eV)), a sputter source (Ar^+^), an ion energy of 1 kV, a sputter area of 1 × 1 mm, a sputter rate of 0.3 nm/s for SiO_2_, and a beam size of 100 µm.

## 3. Results and Discussion 

**Figure 1:**
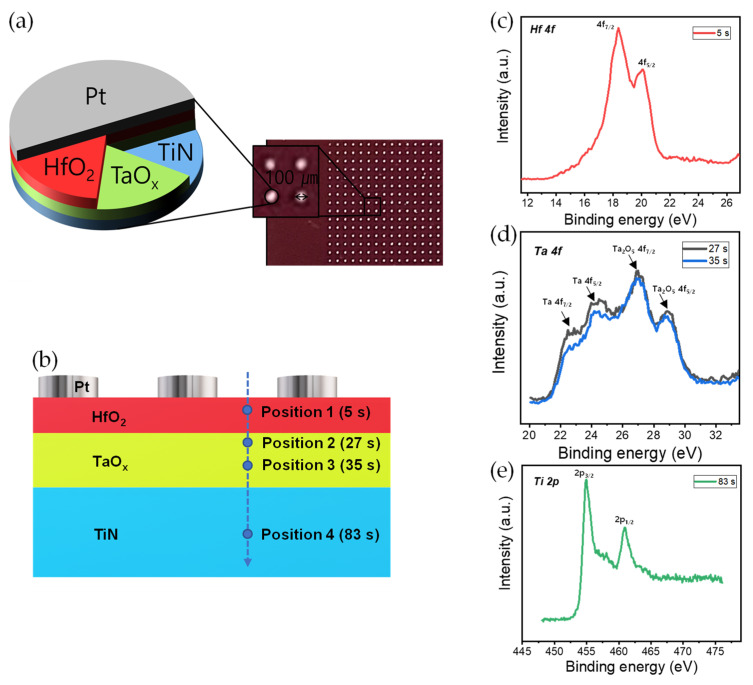
(**a**) Schematic of Pt/HfO_2_/TaO_x_/TiN device stack and top view of the device; (**b**) cross-section image for XPS depth profile. XPS spectra of (**c**) Hf 4f, (**d**) Ta 4f, and (**e**) Ti 2p.

Figure 1a shows schematics of the Pt/HfO_2_/TaO*_x_*/TiN device. The stack of the device is defined through a circular dot with a diameter of 100 µm. The XPS depth profile mode of the HfO_2_/TaO*_x_*/TiN layers, except for the Pt top electrode, was taken to obtain the chemical information (Figure 1b and Figure S1). Figure 1c shows the XPS spectra of Hf 4f for the HfO_2_ layer as the first insulator at 5 s (position 1). Two peaks of the Hf 4f_7/2_ and Hf 4f_5/2_ core-level binding energies are located at 18.4 eV and 20 eV, respectively. This indicates that Hf is fully oxidized for the nearly stoichiometric HfO_2_ film that was deposited by ALD [31]. Figure 1d shows the XPS spectra of Ta 4f for TaO*_x_* film as the second insulator layer at 27 s and 35 s (positions 2 and 3, respectively). In addition, the peak binding energies of Ta_2_O_5_ 4f_7/2_ and 4f_5/2_ are respectively located at about 26.6 and 28.8 eV. Further, the peaks for Ta 4f_7/2_ and Ta 4f_5/2_ are detected at about 22.6 and 24.2 eV, respectively, indicating the existence of metallic Ta in the TaO*_x_* film deposited by DC sputtering. The Ta 4f_7/2_ at position 2 (near the HfO_2_ layer) is clearer than that at position 3 (bulk), which suggests the oxygen reduction of TaO*_x_* during the HfO_2_ deposition. In addition, oxygen interchange happens easily during the device operation, such as the set and reset process [32]. Finally, we observe the Ti 2p XPS spectra at 82 s (position 4), as shown in Figure 1e. The peaks of Ti 2p_3/2_ and Ti 2p_1/2_ are respectively centered at about 454.9 eV and 461 eV for the TiN bottom electrode [33].

**Figure 2:**
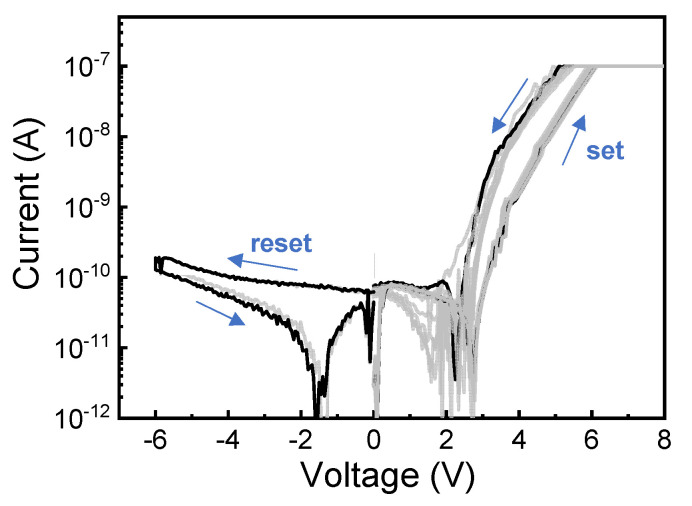
I–V characteristics with self-rectification of Pt/HfO_2_/TaO_x_/TiN device.

Figure 2 shows the self-rectifying resistive switching I–V characteristics with a compliance current (CC) of 100 nA for a Pt/HfO_2_/TaO*_x_*/TiN device. Bipolar resistive switching with self-rectifying behavior can clearly be observed. The results show that the current increases at the same voltage through dual DC sweep from 0 V to 6 V for the set process. By contrast, the resistance is decreased from high-resistance state (HRS) to LRS for the set process. On the other hand, the reset process occurs by dual DC sweep from 0 V to –6 V to make the transition from LRS to HRS. It should be noted that the currents in both LRS and HRS are significantly suppressed at negative bias voltage. In other words, no apparent current decrease is observed during the reset process. This resistive switching behavior is similar to previously reported results on MIIM self-rectifying RRAM devices [20,21,22]. For comparison with the single layer devices, the resistive switching characteristics of the Pt/TaO*_x_*/TiN and Pt/HfO_2_/TiN devices are also checked (Figure S2). The two single layer devices show typical bipolar resistive switching without rectifying characteristics when high CC is applied to the devices. 

**Figure 3:**
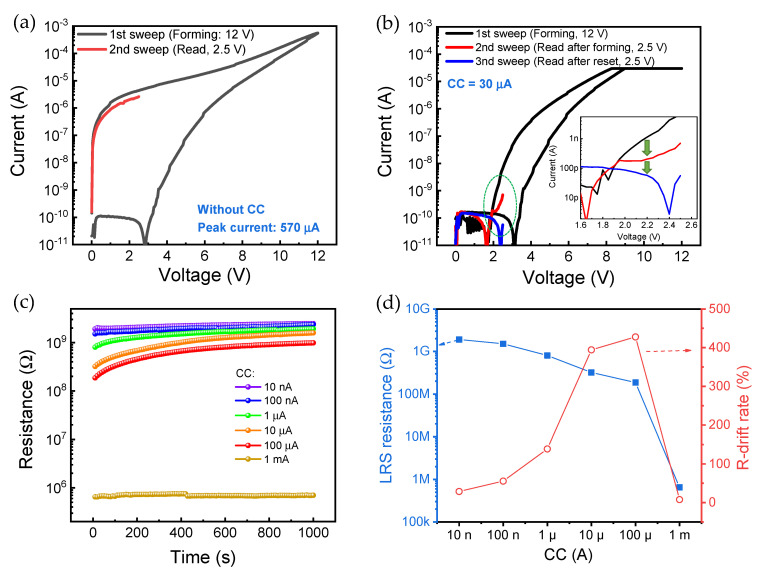
Volatile characteristics of Pt/HfO_2_/TaO*_x_*/TiN device. DC read sweep after forming voltage (**a**) without CC for high current level in the LRS and (**b**) with CC of 30 µA for low current level; (**c**) R-drift characteristics with different CCs from 10 nA to 1 mA; (**d**) LRS resistance and R-drift change as a function of CC.

To verify the volatile and nonvolatile characteristics of the Pt/HfO_2_/TaO*_x_*/TiN memristor device, the DC sweep is first conducted for different LRS states by controlling CC (Figure 3). The forming process is conducted without CC for high current in the LRS (Figure 3a). The current is increased considerably after the forming process with the sweep voltage of 12 V. After a delay of about 30 s, it can be confirmed that the current in the LRS is noticeably decreased in the sweep for reading the current. This indicates that the Pt/HfO_2_/TaO*_x_*/TiN device is not perfectly non-volatile memory and that data are lost over time. Further, the reset process does not occur at high current level in the LRS. This means that only one-time programmable memory can be realized in this case. The non-volatile property of low-level current in the LRS is also checked with CC of 30 µA, and the results are shown in Figure 3b. The current in the LRS is slightly decreased after forming sweep, like in the high LRS current case without CC. Moreover, it is confirmed that the reset process can activate the current reduction. To elucidate the volatile property, we check the retention characteristics at more CC points. Figure 3c shows resistance-drift (R-drift) as a function of time with different CC (10 nA, 100 nA, 1 µA, 10 µA, 100 µA, and 1 mA). It can clearly be observed that LRS resistance is gradually decreased with increasing CC from 10 nA to 100 µA (Figure 3d). However, the LRS resistance is changed drastically at CC of 1 mA, which indicates that the effective filament is formed in both insulators. Here, R-drift is defined as follows:

(1) $$R-\mathrm{drift}\;\mathrm{rate}=\frac{Initial\;resistance\;–\;resistance\;at\;10000\;s}{Initial\;resistance}\;\;\;\;\;\;\;\;\;\;\;\;\;\;\;\;\;$$

The resistance values in the LRS increase with time, and the LRS resistance and the R-drift rate are inversely proportional to each other. In previous studies, the resistive switching mechanism of the Pt/HfO_2_/TaO*_x_*/TiN device was explained by charge trapping/detrapping [20,22]. The conductance is increased when the electron is trapped at defect sites in the insulators, and it is decreased when detrapping occurs. The R-drift rate increases as the CC increases in the charge trapping dominant switching mechanism region (10 nA–100 µA). The above result means that the higher the conductance is increased by charge trapping, the faster the resistance drifts. The R-drift rate is very low when CC is 1 mA, implying that the resistive switching at CC of 1 mA is substantially different than those at lower CC regions.

**Figure 4:**
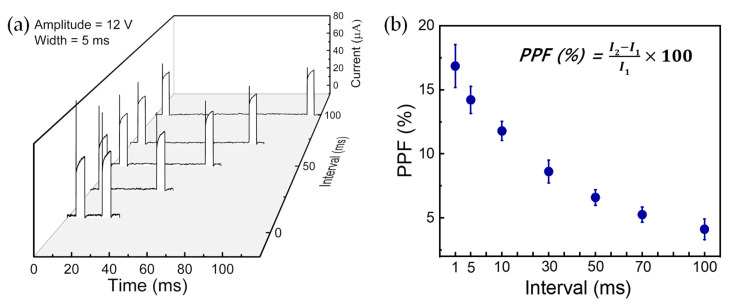
PPF characteristics of the Pt/HfO_2_/TaO*_x_*/TiN device. (**a**) I–V characteristics with different interval times. (**b**) Statistical distribution of PPF as a function of interval time.

To use the R-drift characteristics, we can apply short-term memory. The neural facilitation known as PPF is explained by the short-term plasticity. In biological synapses, PPF is a phenomenon that can increase synaptic weight by controlling the interval between sequential pulse stimuli; specifically, the postsynaptic potential is increased as the time between the pre-impulse and post-impulse is reduced. The paired pulses (pulse amplitude of 12 V, pulse width of 5 ms) are applied on the Pt/HfO_2_/TaO*_x_*/TiN device in which the interval time between pre-pulse and post-pulse is varied (1 ms, 5 ms, 10 ms, 30 ms, 50 ms, 70 ms, and 100 ms), as shown in Figure 4a. A statistical analysis is used to quantitatively determine the trend of the change rate between the first and second pulses in two sequential PPF pulses (Figure 4b). Here, the PPF index is calculated by the current at the middle point of the first pulse (*I*_1_) and the current at the middle point of the second pulse (*I*_2_).

(2) $$\mathrm{PPF}\;(\%)=\frac{I_{2}-I_{1}}{I_{1}}\times 100$$

The blue bars express the PPF (%) of the Pt/HfO_2_/TaO*_x_*/TiN device; the bar represents the minimum from the maximum while the circle means the average (Figure 4b).

The exponential fitting curve is defined by the following equation:

PPF curve = C_1_·exp(−t/τ_1_) + C_2_·exp(−t/τ_2_) (3)

where C_1_ was 3.13, C_2_ was 7, τ_1_ was 35, and τ_2_ was 3000. The smaller the interval is, the greater the change rate of the second pulse compared to the first pulse; this is similar to having a stable memory when a short stimulation period is applied in biological synapses. On the other hand, as the time interval increases, the change rate of conductance decreases. This is likened to having unstable memories when a long stimulation period is applied in biological synapses. 

**Figure 5:**
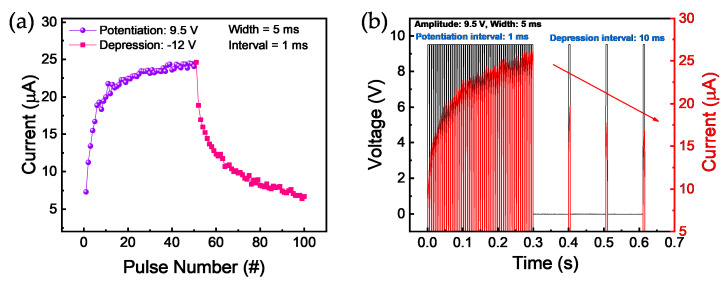
Potentiation and depression characteristics of the Pt/HfO_2_/TaO*_x_*/TiN device. (**a**) Current modulation controlled by set and reset pulses; (**b**) natural depression process that involves increasing the interval time between pulses.

It is desirable to implement multi-level conductance in neuromorphic systems using synaptic devices such as RRAM. To emulate a biological synapse, the method of controlling conductivity could be closer to an analog process than a digital process. The Pt/HfO_2_/TaO*_x_*/TiN memristor device has a certain volatile characteristic, but long-term plasticity, including potentiation and depression, is achieved by pulse mode without a long delay between each pulse. It is understood to involve a temporary increase and decrease in conductance because strong continuous pulses are applied for the Pt/HfO_2_/TaO*_x_*/TiN device. Figure 5a shows the potentiation and depression curve obtained by controlling pulse polarity. Potentiation and depression are measured by applying values of 9.5 V and −12 V with a pulse width of 5 ms for synaptic properties, respectively. The identical pulses continue to be applied to the devices for the set/read and reset/read at the same time for the potentiation and depression, respectively. Next, we demonstrate potentiation and depression using another method. Figure 5b shows a natural depression implemented by short-term plasticity rather than by artificial depression using pulse stimulation. The current is increased by applying constant and identical pulse inputs with the amplitude of 9.5 V, the width of 5 ms, and the interval time of 1 ms. As a result, the current gradually decreases when the same pulse with a relatively larger interval of 10 ms is placed. It is described as a type of short-term memory in which the current decreases as the frequency of stimulation decreases. This is a spontaneous collapse phenomenon that occurs in the weak set process, as we already discussed the strength of LRS, which is controllable by CC. These results suggest that both long-term memory and short-term memory can be implemented, depending on the spacing of the input voltage pulse stimuli.

## 4. Conclusions

In summary, the results prove that the Pt/HfO_2_/TaO*_x_*/TiN memristor device has self-rectifying resistive switching I–V characteristics. First, the device stacks deposited by sputtering and atomic layer deposition are well confirmed by XPS analysis. We also thoroughly reveal the volatile property by DC voltage sweep and retention test. In addition, the degree of data decay can be adjusted by setting the LRS current level by compliance current. Further, the device has characteristics of current decay that can mimic neuronal facilitation for short-term memory. The multi-level conductance adjustment while maintaining self-rectifying characteristics is achieved by placing consecutive pulse inputs for potentiation and depression. Moreover, the natural depression is emulated by increasing the pulse interval time. These bio-synaptic features, such as STP and LTP, are successfully imitated in the Pt/HfO_2_/TaO*_x_*/TiN memristor device. Altogether, this indicates that the Pt/HfO_2_/TaO*_x_*/TiN memristor could be used for the artificial synapses in neuromorphic computing.

## Supplementary Materials

The following are available online at https://www.mdpi.com/2079-4991/10/11/2159/s1, Figure S1: XPS depth profile. Figure S2: I–V characteristics of the Pt/HfO_2_/TiN and Pt/TaO*_x_*/TiN devices.
