# Supplementary material for: Self-Rectifying Resistive Switching and Short-Term Memory Characteristics in Pt/HfO2/TaOx/TiN Artificial Synaptic Device
Source: Nanomaterials (Basel). 2020 Oct 29;10(11):2159. doi: 10.3390/nano10112159 (PMC7693614; doi:10.3390/nano10112159)
Supplement: Supplementary file 1 [file nanomaterials-10-02159-s001.pdf]

Supplementary Materials for Self-Rectifying Resistive Switching and Short-Term Memory Characteristics in Pt/HfO_2/TaO_x/TiN Artificial Synaptic Device

Hojeong Ryu and Sungjun Kim *

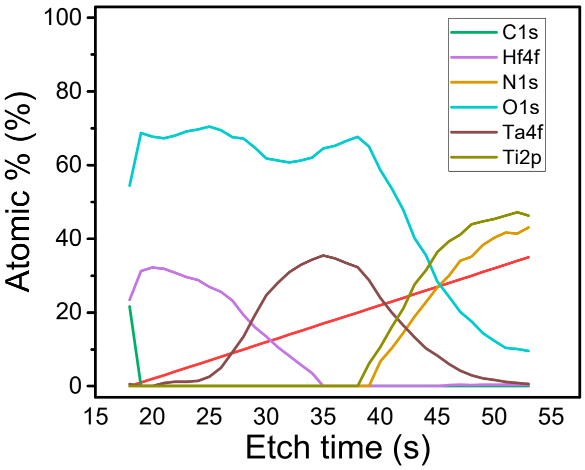

Figure S1. XPS atomic % depth profile of HfO_2/TaO_x/TiN device.

(a)

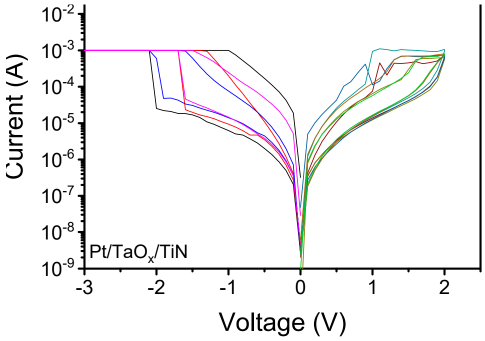

(b)

Figure S2. I-V curves of (**a**) Pt/TaO_x/TiN device and (**b**) Pt/HfO_2/TiN devices.
